# Possible new mechanisms of primary drug resistance in NSCLC with EGFR mutation treated with Osimertinib

**DOI:** 10.1002/iub.70002

**Published:** 2025-02-05

**Authors:** Lujing Shao, Tong Li, Xinyan Jia, Xinyu Zhang, Qi Li, Chunyan Dong

**Affiliations:** ^1^ Department of Oncology, East Hospital Affiliated to Tongji University Tongji University School of Medicine, Tongji University Shanghai China; ^2^ Department of Oncology, Postgraduate Training Base of Jinzhou Medical University Shanghai East Hospital Shanghai China; ^3^ Ji'an Central People's Hospital (Ji'an Hospital of Shanghai East Hospital) Ji'an China

**Keywords:** EGFR mutation, NSCLC, Osimertinib, Pemetrexed, primary drug resistance

## Abstract

In this study, a patient with lung adenocarcinoma harboring an EGFR mutation exhibited primary resistance to the targeted EGFR inhibitor Osimertinib after 2 months of treatment. As the disease advanced, further genetic analysis revealed the emergence of additional mutations in ARID1A, NTRK1, and ZRSR2, alongside the existing EGFR mutation. Subsequent treatment with Pemetrexed resulted in a significant reduction in liver metastases. Protein mass spectrometry sequencing and immunohistochemical analysis collectively indicated that the PI3K/mTOR pathway mediates the mechanism through which these gene mutations confer primary drug resistance. Evidence demonstrates that the co‐occurrence of EGFR and ARID1A mutations diminishes the efficacy of EGFR tyrosine kinase inhibitors (EGFR TKIs). Consequently, it is hypothesized that mutations in NTRK1 and ZRSR2, which are implicated in the PI3K/mTOR pathway, contribute to the primary resistance observed with Osimertinib treatment. In this case, the illness was effectively managed through prompt adjustments to the treatment regimen and the rapid administration of chemotherapy drugs. This finding also constitutes the first evidence that mutations in NTRK1 and ZRSR2 are pivotal in the development of primary resistance to Osimertinib. Consequently, it is imperative to conduct genetic testing at the earliest opportunity and modify the treatment plan accordingly.

## 1 INTRODUCTION

Lung adenocarcinoma (LUAD) is the most prevalent form of lung cancer, constituting approximately 40% of cases.^1^ Due to the enhanced efficacy and tolerability of tyrosine kinase inhibitors (TKIs) in the management of non‐small cell lung cancer (NSCLC) relative to traditional cytotoxic chemotherapy, the principal aim of treatment is to precisely identify patients who are likely to benefit from targeted therapeutic interventions.^2^

Osimertinib, originally authorized for the treatment of resistance induced by the T790M mutation to first‐ and second‐generation EGFR‐targeted therapies, demonstrates a significant objective response rate of 80% and a median progression‐free survival (PFS) of 18.9 months. Primary drug resistance is relatively rare and is predominantly associated with the presence of B‐cell lymphoma 2‐like 11 (BIM) deletion polymorphism and EGFR Exon 20 insertions.^3^

Here, we present the initial instance of a non‐small cell lung cancer (NSCLC) patient with an EGFR mutation who also harbored both NTRK1 and ZRSR2 mutations, which are recognized as significant factors in the development of primary resistance to Osimertinib through further validation.

## 2 CASE PRESENTATION

A 46‐year‐old Chinese female experienced symptoms of chest tightness and shortness of breath following physical exertion in June 2022. Fatigue was also reported, while fever, cough, expectoration, and discomfort were absent. A chest CT scan revealed the presence of a sizable lesion. After undergoing left thoracic puncture and drainage surgery, the exfoliated cell pathological examination identified atypical proliferative cells derived from epithelial tissue, suggesting adenocarcinoma. Subsequent gene testing of the pleural effusion detected EGFR (12.10%) exon19, c.2235_2249del, p.E 746_A750del (E19del).

**FIGURE 1:**
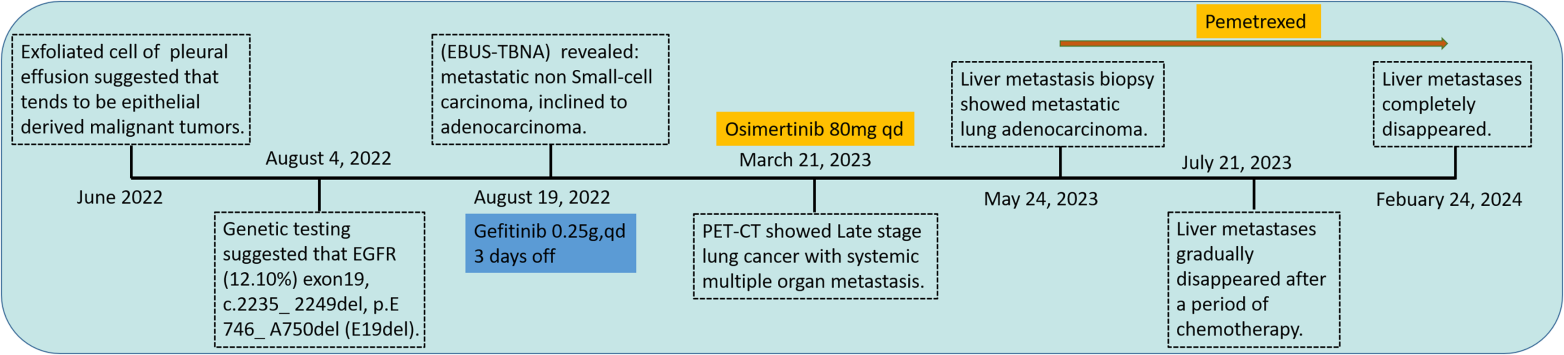
Overview of patient disease progression.

In order to obtain a more comprehensive diagnosis and treatment, she was referred to Shanghai Pulmonary Hospital where a PET‐CT examination revealed the presence of pulmonary MT accompanied by multiple systemic metastases. Additionally, an ultrasound bronchoscopy guided trans bronchial needle aspiration was performed. After comprehensive consideration of various examinations and genetic testing, the patient was advised to take Gefitinib orally for the treatment of metastatic non‐small cell carcinoma with adenocarcinoma tendencies (in the mediastinal lymph nodes). However, the patient refused this recommendation (Figure 1).

**FIGURE 2:**
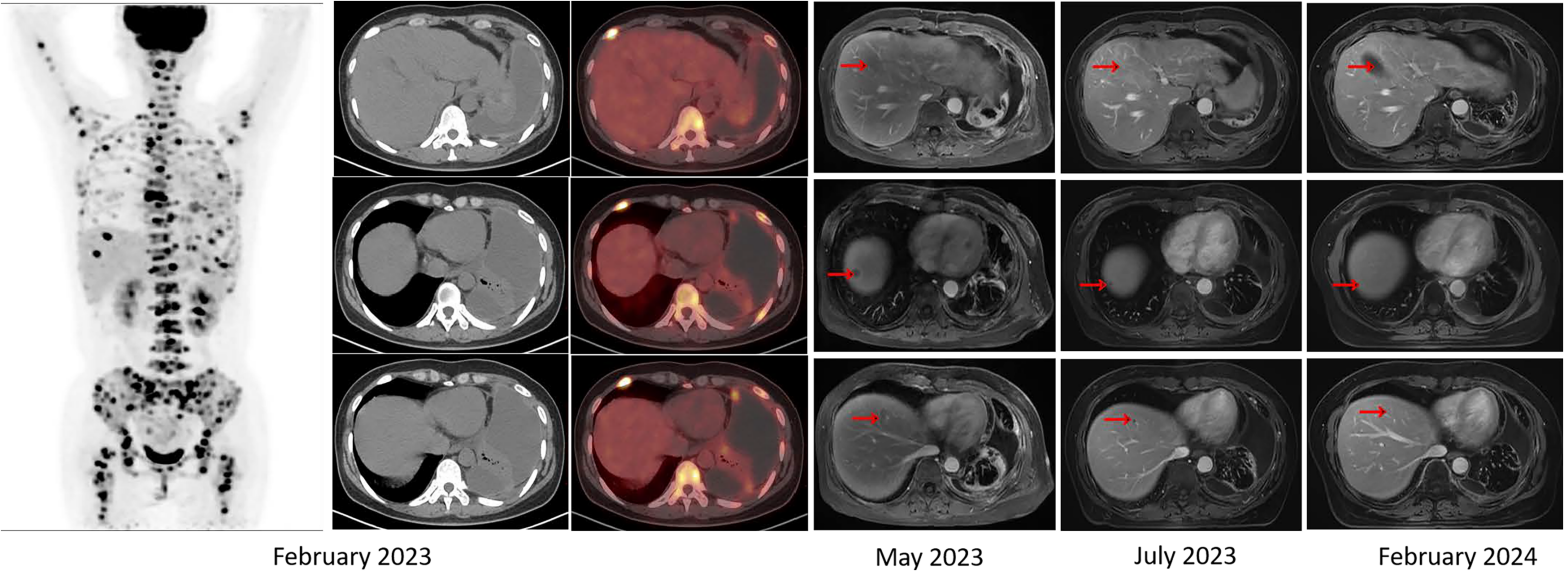
Comparison of liver imaging before and after tumor liver metastasis. In February 2023, PET/MRI results showed that the liver was in good condition and no metastatic tumors were found (in the left column). In May 2023, enhanced CT results indicated multiple liver metastases (in the third column from right to left). In July 2023, liver metastases gradually disappeared after a period of chemotherapy (in the second column from right to left). In February 2024, liver metastases completely disappeared (in the right column).

In March 2023, the patient felt chest and back pain without limited mobility. She visited our department for another PET‐CT examination, and the results were the same as before, with a new widespread bone metastasis throughout the body (Figure 2, left column). After comprehensive consideration of various examinations and genetic testing, the patient was given anti‐bone metastasis treatment combined with Osimertinib Targeted therapy from March 23, 2023.

**FIGURE 3:**
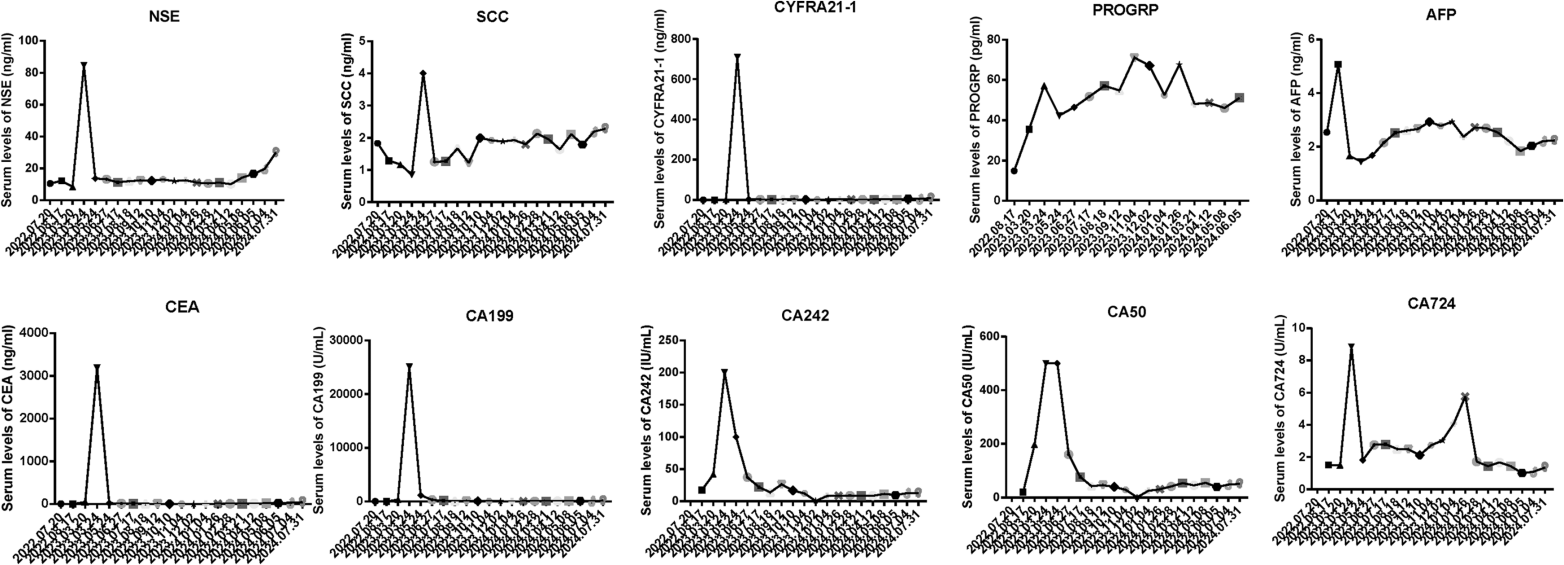
Time‐course changes of various tumor markers in patients. Each panel represents the dynamic changes of a specific tumor marker over time. Top row, from left to right: The first panel shows the changes of NSE (Neuron‐Specific Enolase). The second panel shows the changes of SCC (Squamous Cell Carcinoma Antigen). The third panel shows the changes of CYFRA 21‐1 (Cytokeratin 19 Fragment). The fourth panel shows the changes of ProGRP (Pro‐Gastrin‐Releasing Peptide). The fifth panel shows the changes of AFP (Alpha‐Fetoprotein). Bottom row, from left to right: The first panel shows the changes of CEA (Carcinoembryonic Antigen). The second panel shows the changes of CA19‐9 (Carbohydrate Antigen 19‐9). The third panel shows the changes of CA125 (Carbohydrate Antigen 125). The fourth panel shows the changes of CA15‐3 (Carbohydrate Antigen 15‐3). The fifth panel shows the changes of CA72‐4 (Carbohydrate Antigen 72‐4). The *x*‐axis represents time, and the *y*‐axis represents the concentration of the tumor markers.

On May 24, 2023, the patient was admitted to the hospital for reexamination and evaluation. The enhanced CT showed multiple liver metastases (Figure 2, middle column with red arrow). The serum oncological markers, including NSE, SCC, CYFRA21‐1, PROGRP, CA199, CA242, CA50, and CA724, fluctuated, but all showed an increasing trend since July 2022. At the same time, AFP and CEA have slightly decreased compared to before, while still significantly higher than the normal range (Figure 3). To further clarify the liver metastases, the patient received ultrasound guided liver puncture. Postoperative pathological findings: (Liver puncture) Poorly differentiated adenocarcinoma, combined with IHC results, considering lung origin. Immunohistochemical results: CK7 (+), TIF1 (+), Napsin A (+), Ki67 (50%, +), CK19 (+), PD‐L1 (clone E1L3N, TPS >50%), CD99 (−), Villin (−).

**FIGURE 4:**
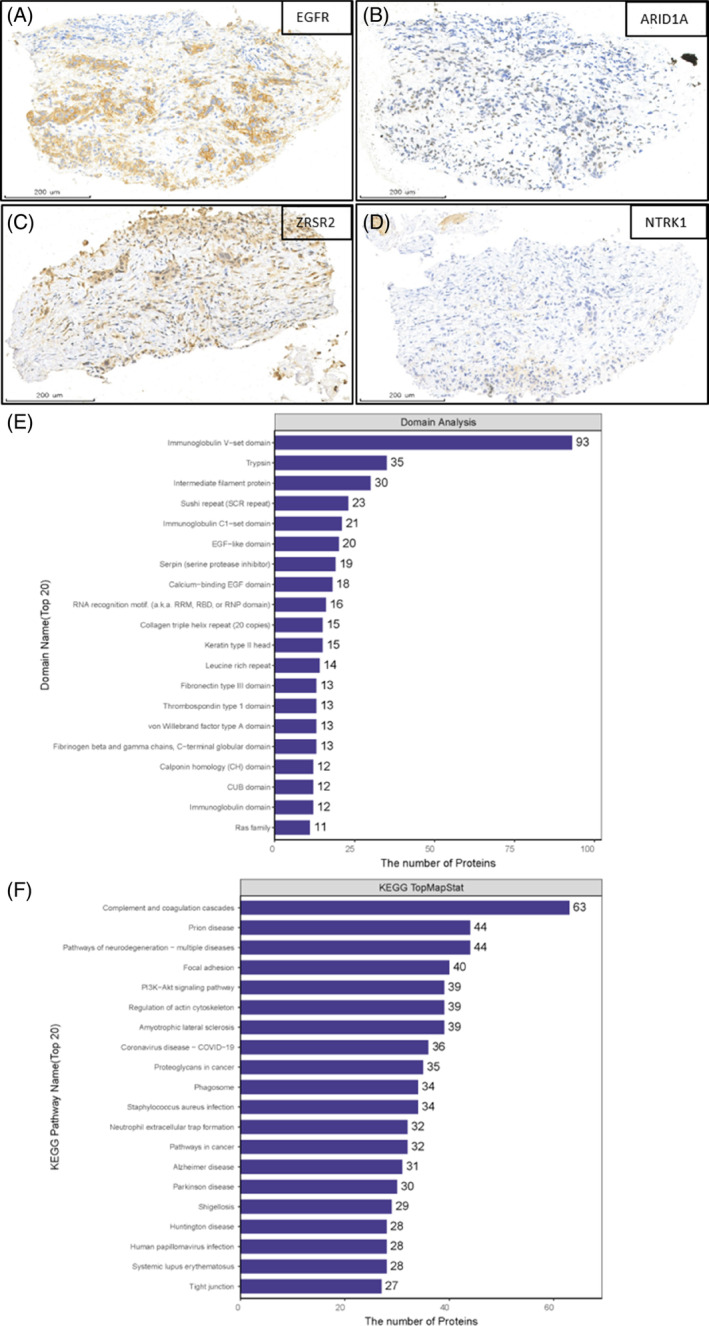
Correlation analysis of genes and biological pathways involved. (A–D) Immunohistochemical staining of EGFR, ARID1A, ZRSR2, TRKA (brown represents positive expression, and the darker the brown, the stronger the positive expression). (E) Differential expression protein domain analysis histogram. (F) Statistical map of KEGG pathway annotations for differentially expressed proteins (Top20).

To explore the potential mechanism of rapid drug resistance in this patient, we obtained liver metastatic tumor tissue through puncture with the patient's consent. Through Genetic testing, besides EGFR mutation, we found ARID1A‐R1989* (4.00%), NTRK1‐R220W (45.60%), combined with ZRSR2‐A95T (50.80%). Immunohistochemical staining was performed on exfoliated cells in pleural effusion. Due to the lack of samples from the patient, this is impossible to compare it with the control group. Only the protein expression in the exfoliated cells from pleural effusion in this case is presented (Figure 4A–D). Further functional testing analysis found that EGF domain is enriched, and differential proteins participate in multiple biological processes; protein interaction results confirm that multiple proteins on the PI3K signaling pathway can bind together in this case and exhibit strong biological functions in tumors (Figure 4H,I).

**FIGURE 5:**
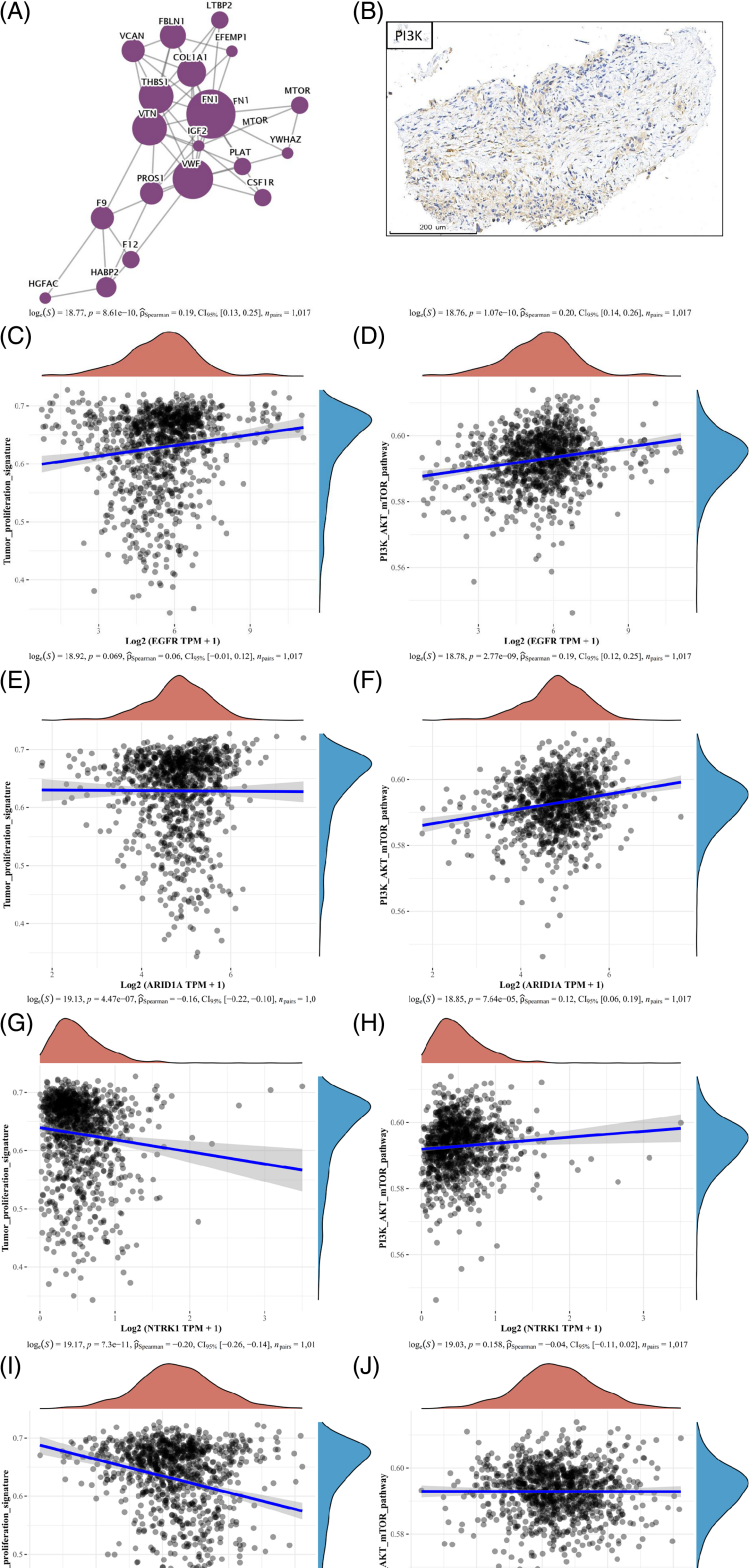
The correlations between individual gene and pathway score. (A) Protein interaction network structure diagram. (B) Immunohistochemical staining of PI3K and surface markers of immune cells (brown represents positive expression, and the darker the brown, the stronger the positive expression). (C–J) The correlations between individual gene and pathway score were analyzed with Spearman. The abscissa represents the distribution of the gene expression, and the ordinate represents the distribution of the pathway score. The density curve on the right represents the trend in distribution of pathway immune score, the upper density curve represents the trend in distribution of the gene expression. The value on the top represents the correlation p value, correlation coefficient and correlation calculation method.

In addition, through in‐depth discussion and analysis of differential proteins (all were compared with public data), it was found that the PI3K/mTOR signaling pathway plays an important role in this primary drug‐resistant case (Figure 5A,B). It is interesting to note that EGFR, ARID1A, and ZRSR2 have a positive correlation with the PI3K/mTOR pathway (*p* < .05). Moreover, we studied the correlation between gene expression and pathway scores through Spearman correlation analysis from the TCGA database (https://portal.gdc.cancer.gov). We found that EGFR, ARID1A, NTRK1, and ZRSR2, acting alone or in concert, can accelerate the growth and spread of tumors and all be positively correlated with PI3K/AKT (Figure 5C–J).

**FIGURE 6:**
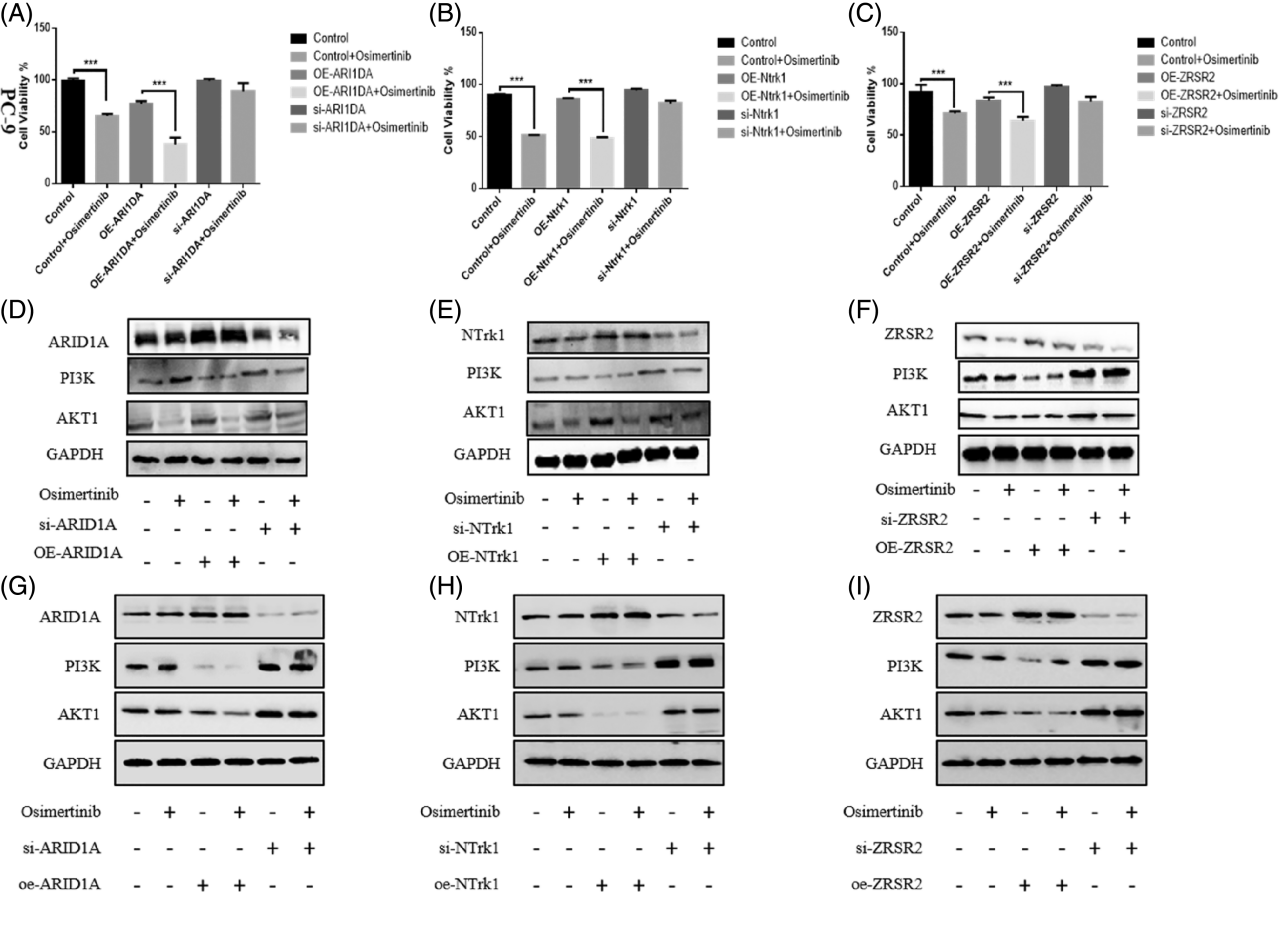
The effect of different degrees of expression of individual genes on the action of Osimertinib. (A–C) Overexpression or knockdown of ARID1A, NTRK1, and ZRSR2 using plasmids, followed by treatment with Osimertinib to detect cell resistance. (D–F) Overexpression or knockdown of EGFR, NTRK1, and ZRSR2 using plasmids, followed by treatment with Osimertinib to detect PI3K‐AKT in PC‐9. (G–I) Overexpression or knockdown of EGFR, NTRK1, and ZRSR2 using plasmids, followed by treatment with Osimertinib to detect PI3K‐AKT inHCC827.

To further validate our findings, using two representative non‐small cell lung cancer cell lines, PC‐9 and HCC827, we compared the expression levels after gene mutations (Figures S1 and S2, Supporting Information). Then we overexpressed and knocked down NTRK1, ZRSR2, and ARID1A, and treated them with sufficient amounts of Osimertinib. We found that the decreased expression of these genes can to some extent affect Osimertinib resistance (Figure 6A–C), thereby promoting the expression of PI3K‐AKT (Figure 6D–F).

## 3 MATERIALS AND METHODS

### 3.1 Cell culture

PC‐9 and HCC827 cells were purchased from the Cell Resource Center of Shanghai Academy of Biological Sciences, Chinese Academy of Sciences. All cell lines were tested and confirmed to be free of mycoplasma contamination. HCC827 and PC‐9 cells were cultured in RPMI with 10% FBS at 37°C in a humidified incubator (with 5% CO2). HCC827 and PC‐9 cells were transfected with plasmids for 48 h. The plasmids were used for the knockdown of EGFR (si‐EGFR), NTRK1 (si‐NTRK1), ZRSR2 (si‐ZRSR2) and the overexpression of EGFR (OE‐EGFR), NTRK1 (OE‐NTRK1), ZRSR2 (OE‐ZRSR2). Meanwhile, corresponding mutant plasmids were transfected above cell lines. After the plasmid transfection for 24 h, the cells were treated with Osimertinib at a concentration of 20 nM for 72 h. All cell culture experiments were repeated three times, with three duplicates per group each time.

### 3.2 Plasmid construction

The construction of knockdown plasmids for EGFR, NTRK1, and ZRSR2, abbreviated as si‐EGFR, si‐NTRK1, and si‐ZRSR2, was achieved using the pLKO vector. Overexpression plasmids for EGFR, NTRK1, and ZRSR2, abbreviated as OE‐EGFR, OE‐NTRK1, and OE‐ZRSR2, were constructed using the pLVX vector. At the same time, the corresponding mutant plasmids were constructed using the pLVX vector. The sequences of plasmids and primers are detailed in Data S1. The control group was transfected with an empty vector.

### 3.3 Immunohistochemistry

The sedimented cells in pleural effusion were embedded in paraffin, sliced, and subjected to dewaxing, hydration, antigen repair, and serum blocking. The antibodies EGFR, NTRK1, ZRSR2, and ARID1A were incubated overnight, followed by the addition of secondary antibodies. The expression of the antibodies was observed under a microscope using DAB staining. A darker brown color indicates a stronger protein expression. Finally, the positive rates were statistically analyzed using Image J software. The tissue chip utilizes the PatternQuant module in Quant Center 2.3 analysis software to differentiate between brown areas (including positive areas) and blue areas. The tissue area was defined by two parts, namely the Mask Area. HistoQuant is applied to the lower level of the brown area to calculate the area and grayscale of the positive areas, and the optical density is determined based on the grayscale.

### 3.4 Western immunoblotting

All cells were homogenized in RIPA buffer (Beyotime Biotechnology). Samples were resolved onto SDS‐polyacrylamide gels (Beyotime Biotechnology) and blotted onto PVDF membranes (Hybond P, GE Healthcare Bio‐Sciences, Pittsburgh, PA). Primary antibodies anti‐NTRK1 (#DF6822, Affinity), anti‐ZRSR2 (PA5‐41797, Thermo), anti‐PI3K (ab302958, Abcam), anti‐AKT (#AF0836, Affinity), and GADPH (#2118, Cell Signaling) were used. Images were acquired using a C‐Digit chemiluminescent Western blot scanner (LI‐COR, Lincoln, NE).

### 3.5 Data collection and analysis

Four gene expression and clinical data of TCGA pan‐cancer data and GTEx were obtained from the UCSC Xena database (https://xenabrowser.net/datapages/). The analysis was conducted by the R v4.0.3 software. PPI network analysis was performed based on GeneMANIA (http://www.genemania.org), which identifies associated genes. Gene Ontology (GO) analysis was performed using EnrichGO function in the R package “clusterProfiler.” Kyoto Encyclopedia of Genes and Genomes (KEGG) analysis was performed using the EnrichKEGG function of the R package “clusterProfiler.”

### 3.6 Spearman correlation analysis

We downloaded STAR‐counts data and corresponding clinical information for LUAD tumors from the TCGA database (https://portal.gdc.cancer.gov). Subsequently, we extracted data in TPM format and performed normalization using the log2 (TPM + 1) transformation. After retaining samples that contained both RNAseq data and clinical information, we finally selected LUAD samples for further analysis. We collected the genes included in the corresponding pathways and then analyzed them using the GSVA package in R software, choosing the parameter method = “ssgsea” for single‐sample gene set enrichment analysis (ssGSEA). Finally, we studied the correlation between gene expression and pathway scores through Spearman correlation analysis.

## 4 DISCUSSION

**FIGURE 7:**
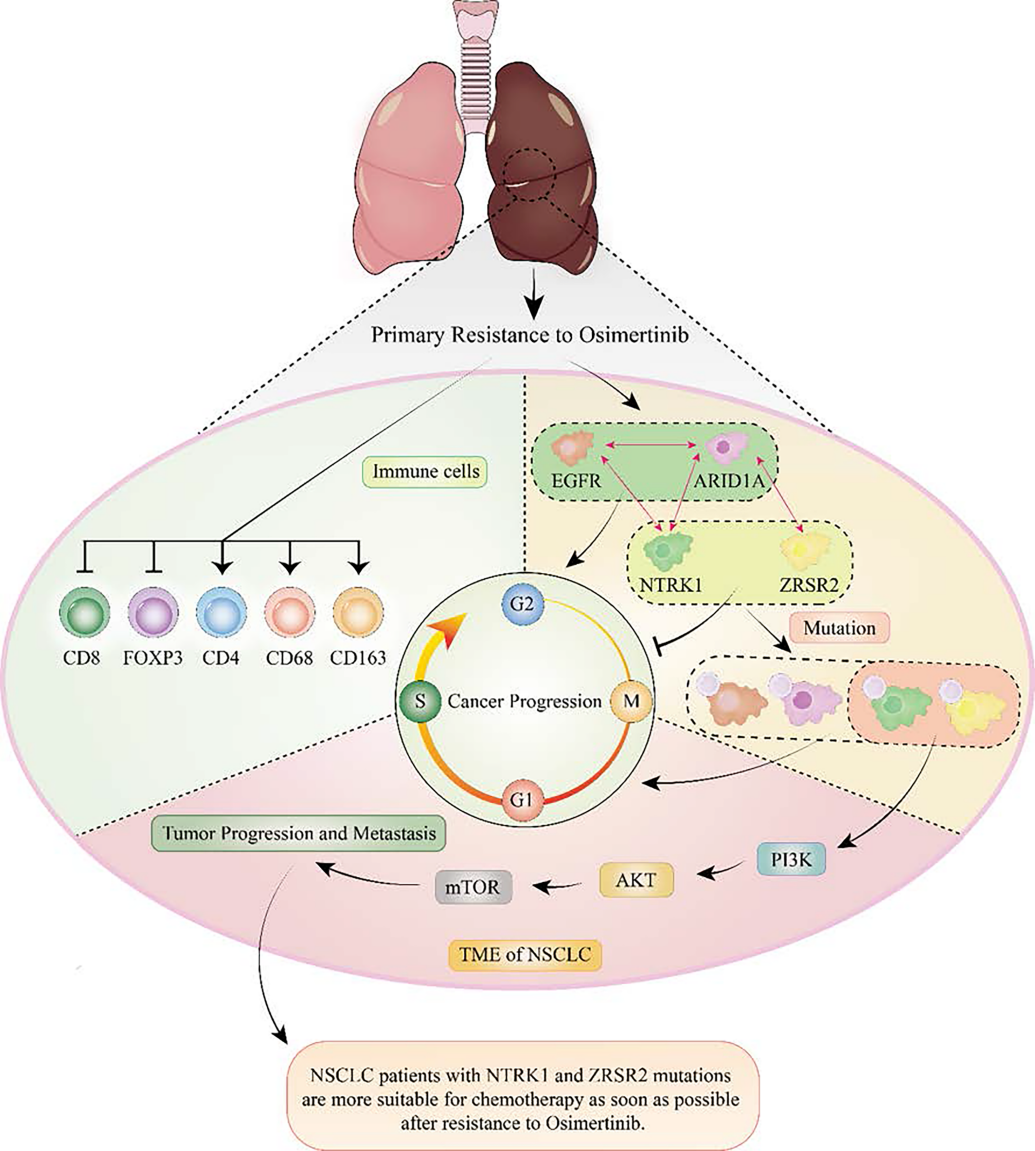
The mechanism of primary drug resistance in non‐small cell lung cancer with ARID1A, NTRK1, and ZRSR2 combined with EGFR mutation treated with Osimertinib.

This is the first reported case of a patient with EGFR mutation in NSCLC who experienced rapid progression after 2‐month treatment with the targeted drug Osimertinib, resulting in multiple liver metastases and primary drug resistance. Secondary genetic testing after progression indicates the presence of ARID1A, NTRK1, and ZRSR2 mutations in addition to the EGFR mutation. Obviously, it was reported that ARID1A is an independent prognostic factor, and co‐mutation of ARID1A and EGFR led to the limited efficacy of EGFR Tyrosine kinase inhibitor (EGFR‐TKIs), but the specific mechanism is unknown.^4^ The treatment regimen was restructured to address the challenge of drug resistance in patients. Following a 2‐month period of administering chemotherapy in conjunction with targeted therapy, there was a marked reduction in liver metastasis, suggesting the efficacy and sustainability of the current treatment protocol (refer to Figure 2, right column, indicated by the red arrow). Given that primary resistance to Osimertinib is relatively uncommon, with a median progression‐free survival (PFS) of 18.9 months, we employed multiple approaches to investigate the potential mechanisms underlying rapid primary drug resistance. Based on these findings, we hypothesize that missense mutations in NTRK1 and ZRSR2 may contribute to primary resistance to Osimertinib in non‐small cell lung cancer (NSCLC) with EGFR mutations through modulation of the PI3K pathway. However, the precise mechanisms underlying this resistance require further investigation (see Figure 7).

The tropomyosin receptor kinase (TRK) family includes TRKA, TRKB, and TRKC proteins, which are encoded by the NTRK1, NTRK2, and NTRK3 genes, respectively. The binding of neurotrophic factors to TRK protein activates downstream signaling cascades through receptor dimerization, phosphorylation, and the induction of PI3K, RAS/MAPK/ERK, and PLCγ pathways.^5^, ^6^ Abnormal TRK pathway, including gene fusion, protein overexpression, and single nucleotide alterations, was associated with the pathogenesis of many types of cancer.^7^ Among them, NTRK gene fusion is commonly seen in breast or salivary gland secretory carcinoma, infantile fibrosarcoma, lung cancer, and colon cancer and is the most effective carcinogenic event to date. Although some studies have identified point mutations or amplifications in the NTRK gene, so far the mutation of the patient has not been proven to be a driving factor for cancer.^8^, ^9^

ZRSR2 is a splicing factor involved in identifying 3′‐intron splicing sites.^10^ Various sequencing studies have identified somatic ZRSR2 mutations in hematological diseases, such as myelodysplastic syndrome (MDS), chronic lymphocytic leukemia (CLL), chronic myelomonocytic leukemia (CMML), or thyroid cancer.^11^, ^12^ However, the role of ZRSR2 mutations in other tumors has not been analyzed yet. The most common changes related to cancer in ZRSR2 are missense and synonymous substitutions (~50%), with only 30% resulting in nonsense substitution or frameshift changes that result in ZRSR2 deletion.^13^, ^14^ Analysis of human ZRSR2 mutations shows that 46% of mutations are located within 10 nucleotides near 3′ ss or 5′ ss, indicating that these mutations may affect the recognition of splice sites by spliceosomes. There was very little research on ZRSR2 mutations in LUAD, and this case can serve as a supplement.

In our study, it was also found that the PI3K signaling pathway plays an important role in this case. Whether the uncommon rapid diffuse progression and primary resistance to Osimertinib are associated with EGFR, ARID1A, NTRK1, and ZRSR2 mutations needs to be further investigated and PI3K signaling pathway may play an important inducing role. Therefore, secondary biopsy and genetic testing are of great significance for revising new treatment plans. Consideration should be given to developing precise targeted therapy for bypass or downstream pathways in combination with Osimertinib.^15^ The relationship between ARID1A expression and immune cell infiltration and immune score in LUAD with EGFR mutation was negatively correlated.^16^ Therefore, ARID1A mutations may not be a reliable predictor of ARID1A protein expression, but understanding the status of ARID1A mutations and the expression may be important for clinical protocol drafting. In addition, the mutation of NTRK1 and ZRSR2 should not be underestimated. Through transcriptomics combined with protein mass spectrometry analysis, it was found that the expression of TRK1 and ZRSR2 was significantly downregulated, which was negatively related to the expression of PI3K/mTOR. Therefore, the primary resistance to Osimertinib reported in this article is extremely complex, including the involvement of multiple genes, missense mutations in NTRK1 and ZRSR2, which would be the first reported drug‐causing factors involved in NSCLC and its mechanism is worth noting.

## 5 CONCLUSIONS

This study suggests that the mechanism underlying primary resistance to Osimertinib may involve additional genetic factors. To substantiate our conclusions and hypotheses, further investigation with a larger sample size is necessary. Despite the relatively low incidence of primary resistance to Osimertinib and the current lack of clarity regarding its mechanism, caution must be exercised in drawing definitive conclusions from a single case. Long‐term follow‐up is essential to identify any delayed effects of mutations on Osimertinib primary resistance.

Currently, it is imperative for physicians to enhance their comprehension of primary drug resistance, engage in regular follow‐up, and perform molecular testing to identify mechanisms of drug resistance and potential genetic mutations. Consequently, this approach enables clinicians to promptly modify treatment plans, thereby improving patient survival rates and prognostic outcomes.

## 6 LIMITATIONS AND ALTERNATIVES

In this case study, the current findings do not yet offer substantial guidance for clinical practice due to several limitations inherent in our research. First, the involvement of multiple genetic testing institutions precludes a definitive determination of whether the observed discrepancies between the two genetic tests are attributable to a novel mutation or variations in detection technology employed during patient treatment. Our inclination is towards the former explanation. Second, the rapid progression of the patient's condition hindered the timely collection of tissue samples prior to the onset of primary drug resistance. A comparative analysis of pre‐ and post‐drug resistance scenarios is currently insufficient, hindering the exploration of underlying causes for primary drug resistance. Additionally, the causal relationship of NTRK1 and ZRSR2 mutations to primary resistance to Osimertinib remains inadequately explored at the molecular level.

## CONFLICT OF INTEREST STATEMENT

The authors declare no conflicts of interest.

## Supporting information


**Data S1.** Supporting Information.

**Figure S1.** Results of supplementary experiments with PC‐9 cells.
**Figure S2.** Results of supplementary experiments with HCC827 cells.

## Data Availability

We have deposited the sequencing data in a publicly accessible database, which can be found at https://ngdc.cncb.ac.cn/?lang=zh. All data generated or analyzed during this study are included in this article. Further inquiries can be directed to the corresponding author.
