# Supplementary material for: Possible new mechanisms of primary drug resistance in NSCLC with EGFR mutation treated with Osimertinib
Source: IUBMB Life. 2025 Feb 5;77(2):e70002. doi: 10.1002/iub.70002 (PMC11796316; doi:10.1002/iub.70002)
Supplement: Supplementary file 2 — Figure S1. Results of supplementary experiments with PC‐9 cells. Figure S2. Results of supplementary experiments with HCC827 cells. [file IUB-77-0-s002.docx]

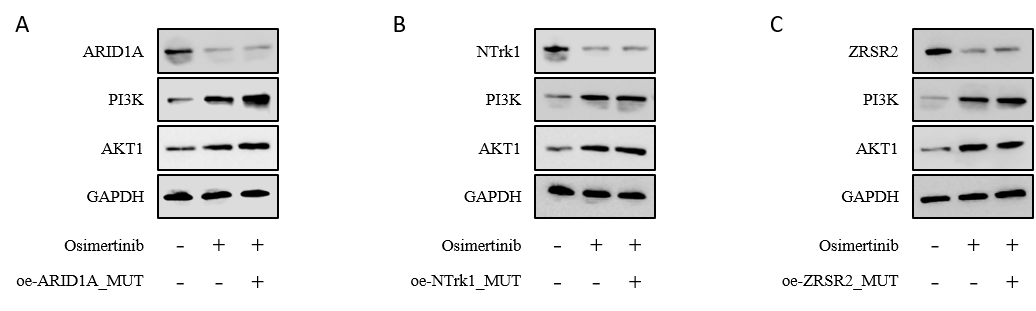


Supplementary Figure 1. Results of supplementary experiments with PC-9 cells.


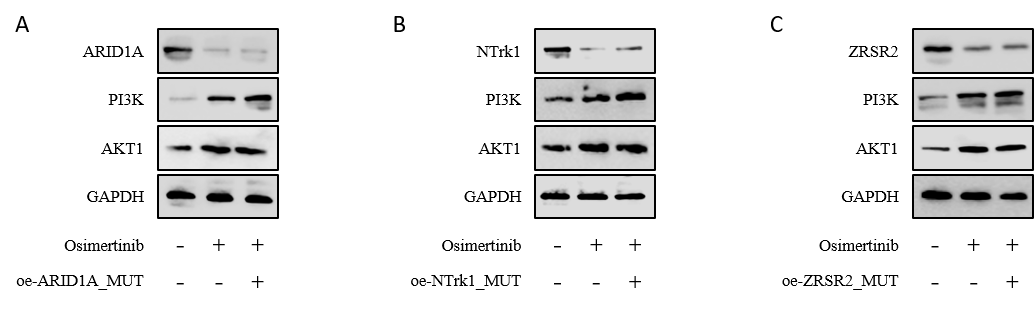


Supplementary Figure 2. Results of supplementary experiments with HCC827 cells.
